# The mitochondrial SIR2 related protein 2 (SIR2RP2) impacts *Leishmania donovani* growth and infectivity

**DOI:** 10.1371/journal.pntd.0005590

**Published:** 2017-05-11

**Authors:** Nimisha Mittal, Rohini Muthuswami, Rentala Madhubala

**Affiliations:** 1 School of Life Sciences, Jawaharlal Nehru University, New Delhi, India; 2 Chromatin Remodelling Laboratory, School of Life Sciences, Jawaharlal Nehru University, New Delhi, India; McGill university, CANADA

## Abstract

**Background:**

*Leishmania donovani*, a protozoan parasite is the major causative agent of visceral leishmaniasis. Increased toxicity and resistance to the existing repertoire of drugs has been reported. Hence, an urgent need exists for identifying newer drugs and drug targets. Previous reports have shown sirtuins (Silent Information Regulator) from kinetoplastids as promising drug targets. *Leishmania* species code for three SIR2 (Silent Information Regulator) related proteins. Here, we for the first time report the functional characterization of SIR2 related protein 2 (SIR2RP2) of *L*. *donovani*.

**Methodology:**

Recombinant *L*. *donovani* SIR2RP2 was expressed in *E*. *coli* and purified. The enzymatic functions of SIR2RP2 were determined. The subcellular localization of *Ld*SIR2RP2 was done by constructing C-terminal GFP-tagged full-length *Ld*SIR2RP2. Deletion mutants of *LdSIR2RP2* were generated in *Leishmania* by double targeted gene replacement methodology. These null mutants were tested for their proliferation, virulence, cell cycle defects, mitochondrial functioning and sensitivity to known SIR2 inhibitors.

**Conclusion:**

Our data suggests that *Ld*SIR2RP2 possesses NAD^+^-dependent ADP-ribosyltransferase activity. However, NAD^+^-dependent deacetylase and desuccinylase activities were not detected. The protein localises to the mitochondrion of the promastigotes. Gene deletion studies showed that Δ*Ld*SIR2RP2 null mutants had restrictive growth phenotype associated with accumulation of cells in the G2/M phase and compromised mitochondrial functioning. The null mutants had attenuated infectivity. Deletion of *Ld*SIR2RP2 resulted in increased sensitivity of the parasites to the known SIR2 inhibitors. The sirtuin inhibitors inhibited the ADP-ribosyltransferase activity of recombinant *Ld*SIR2RP2. In conclusion, sirtuins could be used as potential new drug targets for visceral leishmaniasis.

## Author summary

Sirtuins are present in most organisms, including plants, bacteria, and animals. They play a vital role in promoting an organism’s health and survival. These proteins are involved in the regulation of several functions in eukaryotic cells, including transcriptional repression, recombination, cell cycle, cellular responses to DNA-damaging agents, and longevity. Sirtuins are known to be involved in regulation of vital cellular processes. Hence, they have been proposed as promising targets for the development of antiparasitic drugs. *Leishmania donovani*, a protozoan parasite that causes visceral leishmaniasis is known to express three sirtuins; SIR2RP1, SIR2RP2, and SIR2RP3. We have worked on the functional characterization of the SIR2RP2 protein from *L*. *donovani* in this study. We report that the SIR2RP2 is an NAD^+^-dependent ADP-ribosyltransferase. This protein is present in the mitochondrion of the promastigotes and deletion of both copies of the gene caused reduced growth, compromised mitochondrial functioning and cell cycle arrest in the transgenic parasites. The transgenic parasites also had reduced infectivity. Deletion of *Ld*SIR2RP2 resulted in increased sensitivity of the parasites to the known sirtuin inhibitors. Furthermore, the sirtuin inhibitors were found to inhibit the ADP-ribosyltransferase activity of *Ld*SIR2RP2 thus, indicating that parasitic sirtuins can be exploited as drug targets for antileishmanial chemotherapy.

## Introduction

Acetylation and deacetylation of proteins have recently emerged as a major post-translational modification [1]. Initially described for N-terminal tails of histones [2], reversible acetylation of proteins in the cytoplasm and in the mitochondria, suggest a central role for acetylation in regulatory mechanisms within and outside the nucleus of the cells [3].

The Silent Information Regulator (SIR2), the founding member of the family of sirtuins, was originally described as a regulator of transcriptional silencing of mating-type loci, telomeres and ribosomal DNA [4], and also involved in the lifespan extension of yeast [5]. Since their discovery, SIR2-like genes, known as sirtuins, have been studied in most organisms, including plants, bacteria, and animals, where they play a vital role in promoting an organism’s health and survival [6]. All sirtuins are characterized by a core domain of ~250 amino acids that is highly conserved among different organisms [7]. They were first described to have NAD^+^-dependent deacetylase activity, that consumes the cofactor nicotinamide adenine dinucleotide (NAD^+^), yielding nicotinamide, O-acetyl ADP ribose (AADPR), and the deacetylated substrate [8, 9].

Bacterial and archaeal genomes express one or two sirtuins, but eukaryotes usually have multiple sirtuins, like yeast has Hst1–4 in addition to Sir2p, and humans have seven sirtuins named SIRT1–7 [10], showing a discrete pattern of subcellular localization. However, being present in large number in different cellular compartments, other novel enzymatic activities for sirtuins have also been described like ADP-ribosyltransferase activity involving the transfer of a single ADP-ribosyl group from NAD^+^ to proteins [11] and NAD^+^-dependent desuccinylase and demalonylase activity [12]. ADP-ribosylation activity is known to be present in yeast SIR2, the human SIRT2, the mouse SIRT6, the *Trypanosoma brucei* SIR2RP1, *Leishmania infantum* SIR2RP1 and the *Plasmodium falciparum* SIR2 [11, 13–15]. Desuccinylation and demalonylation activity has only been described for SIRT5 of human [12]. In summary, the evidence emerging from the literature is that the SIR2 proteins regulate the structure/function of multiple targets within the cell, by deacetylation, ADP-ribosylation and desuccinylation/demalonylation of specific lysine residues.

Parasite sirtuins are distributed in all the phylogenetically defined sirtuin classes [16]. Sirtuins of protozoan parasites have both the canonical and atypical activities that contribute to both conserved and apparently unique functions. *T*. *brucei* SIR2RP1, a nuclear protein that co-localizes with telomeric sequences and mini-chromosomes and has both deacetylase and ADP ribosylase activity, is known to be involved in DNA repair [13]. *T*. *cruzi* lacks SIR2RP2 but expresses SIR2RP1 and SIR2RP3 that have been found to be essential for the proliferation of the parasite, host- parasite interplay and differentiation among life cycle stages [17]. Besides this, *Tc*SIR2RP3 has been studied as a possible target for the treatment of Chagas’ disease [18].

The parasite *Leishmania donovani* is a protozoan parasite, the major causative agent of visceral leishmaniasis [19]. The disease is fatal if left untreated. The parasite has a digenetic life cycle which alternates between mammalian immune cells and gut of insect vector, phlebotomine sand flies [20]. The current therapies are inadequate because of the increasing resistance to the currently used drugs and their serious side effects. Hence, an urgent need exists to develop new chemotherapeutic targets and agents against Leishmaniasis.

*Leishmania* parasites are known to express three sirtuins; SIR2RP1, SIR2RP2, and SIR2RP3. Out of the three, only SIR2RP1 has been characterized in *L*. *major* and *L*. *infantum* wherein it was found to be present in the cytoplasmic granules and indispensable for parasite survival [21, 22]. It was found to have both NAD^+^-dependent deacetylase and ADP- ribosyltransferase activities unrelated to epigenetic silencing. The other two sirtuins, SIR2RP2 and SIR2RP3, have not yet been characterized.

Here, we for the first time report the functional characterization of an SIR2RP2 protein from *L*. *donovani*. SIR2RP2 is an NAD^+^-dependent ADP-ribosyltransferase. SIR2RP2 localizes to the mitochondrion of the promastigotes. Gene deletion mutations were attempted via targeted gene replacement methodology in order to elucidate the physiological role of *Ld*SIR2RP2. Deletion of *Ld*SIR2RP2 from the parasite caused compromised mitochondrial functioning and accumulation of cells in the G2/M phase. The null mutants also had attenuated infectivity. Deletion of *Ld*SIR2RP2 resulted in increased sensitivity of the parasites to the known sirtuin inhibitors.

## Methods

### Materials

All restriction enzymes, DNA-modifying enzymes, and DNA ladders were obtained from New England Biolabs. Plasmid pETM-41 and TEV protease were kindly provided by Dr Amit Sharma (ICGEB, New Delhi). Protein markers were obtained from Thermo Fisher Scientific (USA). Nicotinamide Adenine Dinucleotide [Adenylate-^32^P] (800Ci/mmol) was purchased from American Radiolabeled Chemicals (USA). Ni^2+^-NTA agarose and amylose resin were purchased from Qiagen and New England Biolabs, respectively. Sirtinol, nicotinamide, cambinol, and Ex-527 were obtained from Sigma-Aldrich (USA). SIRT1 Fluorometric Drug Discovery Kit and SIRT5 Fluorometric Drug Discovery Kit were procured from Enzo Life Sciences (USA). Other materials used in this study were of analytical grade and were commercially available.

### Strains and culture conditions

*L*. *donovani Bob* (*Ld*Bob strain/MHOM/SD/62/1SCL2D) was originally obtained from Dr Stephen Beverley (Washington University, St. Louis, MO). Wild-type promastigotes were cultured at 22°C in M199 medium (Sigma-Aldrich, USA) supplemented with 100 units/ml penicillin (Sigma-Aldrich, USA), 100 μg/ml streptomycin (Sigma-Aldrich, USA) and 5% heat-inactivated fetal bovine serum (Biowest). Wild-type (WT) parasites were routinely cultured in media with no drug supplementations, whereas the genetically manipulated *LdSIR2RP2* heterozygotes, in which one allele of *LdSIR2RP2* gene has been replaced either with hygromycin phosphotransferase gene (*LdSIR2RP2/HYG*) or with neomycin phosphotransferase gene (*LdSIR2RP2/NEO*) and null mutants (Δ*LdSIR2RP2)* were maintained in either 200 μg/ml hygromycin or 300 μg/ml paromomycin or both respectively. The Δ*LdSIR2RP2* strain containing pSP72a-zeo-a-*LdSIR2RP2* episome (‘add-back’ mutant cell line) was maintained in 800 μg/ml zeocin, 200 μg/ml hygromycin and 300 μg/ml paromomycin. pSP72a-neo-a-GFP-*LdSIR2RP2* transfected parasites were maintained in 40 μg/ml G418. For characterising the mutant parasites phenotypically, cells were sub-cultured without selection antibiotics prior to experiments.

The mouse monocyte-macrophage-like cell line J774A.1 obtained from ATCC was cultured in RPMI 1640 (Sigma-Aldrich, USA) supplemented with 10% FBS and 100 units/ml penicillin and 100 μg/ml streptomycin at 37°C in humidified CO_2_ incubator.

### Multiple sequence alignment and phylogeny

Sirtuin sequences of *Leishmania* and other kinetoplastids were retrieved from TriTrypDB database [23] and used for sequence analysis. The human sirtuin sequences were obtained from UniProt [24]. Subcellular localization prediction was made using a web version of WoLF PSORT [25]. Phylogenetic analysis was performed using MUSCLE [26] and Unrooted software. Multiple sequence alignment of these sequences was generated using a standalone version of CLUSTALW [27] using default parameters. For analyzing the conserved motif patterns and subfamily classification of the kinetoplastid sequences, multiple sequence alignment of only the kinetoplastid sequences was generated.

### Cloning, expression and purification of recombinant *Ld*SIR2RP2

The gene for *LdSIR2RP2* was amplified by PCR using a forward primer with a flanking *Nco*I restriction site (Forward: 5′ AACCATGGCTATGAGGCCGGCGGGGACGATC 3′) and a reverse primer with a flanking *Kpn*I restriction site (Reverse: 5′ AAGGTACCCTAGAGTTGAATCGTCTTGCGGCGGAAG 3′) from *L*. *donovani* genomic DNA. The ~ 963 bp amplicon encompassing the complete ORF of *LdSIR2RP2* gene was cloned into a pETM41 expression vector. The recombinant vector pETM41-*LdSIR2RP2* was transformed into Artic-Express DE3 strain. Expression of recombinant *Ld*SIR2RP2 was induced in mid-exponential phase with 0.1 mM IPTG (isopropyl ß D-thiogalactoside) for 24 h at 10°C. Cells were harvested by centrifugation at 5,000 rpm for 15 min. The bacterial pellet was resuspended in lysis buffer (20 mM Tris-HCl pH 7.2, 200 mM NaCl, 10% glycerol, 10 mM beta-mercaptoethanol, 0.1 mg/ml lysozyme, 2 mM phenylmethylsulfonyl fluoride and protease inhibitor cocktail). The cells were lysed by sonication and cleared by centrifugation at 6,000 rpm for 20 min. The cleared supernatant was applied to pre-equilibrated amylose beads (NEB), and protein was eluted with buffer containing 20 mM Tris–HCl pH 7.2, 200 mM NaCl, 10 mM beta-mercaptoethanol and 2 mM maltose. The tag (MBP with His tag) was removed by incubating with TEV protease at 4°C for 24 h.

### Enzyme activity assays

The NAD^+^-dependent deacetylase activity was estimated by using a commercially available SIRT1 Fluorometric Drug Discovery Kit (Enzo Life Sciences). The enzymatic reaction containing r*Ld*SIR2RP2 was carried out according to the manufacturer’s protocol. Briefly, the recombinant protein was incubated with 50 μM to 1 mM NAD^+^ and 64 μM fluorogenic peptide for 20 min at 37°C followed by incubation in the developer for 45 min at 37°C. The fluorescence was measured with excitation at 360 nm and emission at 460 nm using Varioskan Flash Multimode Reader (Thermo Fisher Scientific). The enzymatic activities were calculated by plotting a standard curve using deacetylated standard available with the kit.

Protein ADP-ribosylation assays were performed as described in [13]. Briefly, the reaction was carried out in a volume of 20 μl containing 2.5 μg of r*Ld*SIR2RP2, 2.5 μCi of [^32^P]NAD^+^ and 5 μg of calf thymus histones (Sigma-Aldrich, USA) or BSA as indicated. The reaction buffer contained 150 mM NaCl, 10 mM dithiothreitol (DTT), 50 mM Tris-HCl pH 8.8. The samples were incubated for 2 h at room temperature. The reactions were terminated by the addition of Laemmli gel loading buffer. The proteins were resolved on a 12% SDS-PAGE gel and visualized with the help of a PhosphorImager (Fujifilm). For quantitative experiments, the reaction products were precipitated with 20% (w/v) trichloroacetic acid (TCA), washed and counted for radioactivity after the addition of scintillation cocktail.

The NAD^+^-dependent lysyl desuccinylase activity was done using a commercially available SIRT5 Fluorometric Drug Discovery Kit (Enzo Life Sciences). The enzymatic reaction having 2.5 μg of r*Ld*SIR2RP2 was carried out according to the manufacturer’s protocol. Briefly, recombinant protein was incubated with 50 μM to 1 mM NAD^+^ and 50 μM fluorogenic peptide for 60 min at 37°C followed by incubation in the developer for 15 min at 37°C. The fluorescence was measured with excitation at 360 nm and emission at 460 nm using Varioskan Flash Multimode Reader (Thermo Fisher Scientific). The enzymatic activities were calculated by plotting a standard curve using desuccinylated standard available with the kit.

### Localisation of *Ld*SIR2RP2 in *L*. *donovani*

Intracellular localisation of *Ld*SIR2RP2 in *L*. *donovani* was detected using pSP72-α-neo-α-GFP-*Ld*SIR2RP2 transfected promastigotes. For the construction of *Ld*SIR2RP2-GFP fusion construct, the 963 bp ORF of *Ld*SIR2RP2 was amplified from *Ld*Bob genomic DNA using sense primer: 5′ AACCATGGCTATGAGGCCGGCGGGGACGATC 3′ and antisense primer 5′ CTCTAGACTAGAGTTGAATCGTCTTGCGGCGGAAG 3′. The restriction sites incorporated in the primers are underlined. The amplicon was cloned into *BamH*1 and *Xba*1 restriction sites of the vector pSP72-α-neo-α-GFP. Correct orientation and sequence fidelity of the inserts was verified by nucleotide sequence analysis. The recombinant vector was transfected by electroporation in wild-type *L*. *donovani* promastigotes according to the standard protocol [28], and the transfectants were selected in the presence of 40 μg/ml G418 (Sigma-Aldrich, USA).

*L*. *donovani*, pSP72-α-neo-α-GFP-*Ld*SIR2RP2 transfected promastigotes were used to detect the cellular distribution of *Ld*SIR2RP2. Log phase promastigotes were incubated with 1 nM MitoTracker red CMXRos (Molecular Probes) diluted in M199 medium for 20 min at 22°C in the dark. The cells were then washed with 1 X PBS and immobilised on poly-L-lysine-coated glass coverslips. Subsequently, the cells were fixed with 4% paraformaldehyde for 30 min, washed and permeabilized in 0.5% Triton X-100-PBS for 5 min. The cellular DNA was then stained with 1 μg/ml of DAPI (Sigma) for 30 min at RT. The coverslips were mounted on glass slides for visualisation.

The cells were imaged by Andor Spinning Disk Confocal Microscope equipped with iXon Ultra 897 EMCCD camera at the required fluorescence excitation and emission wavelengths. The raw images were processed using FV10-ASW 1.7 viewer or Image J software. The co-localization analysis was done using JACoP (Image J).

### Molecular constructs for the replacement of *Ld*SIR2RP2 alleles

Targeted gene replacement strategy was utilized for the inactivation of *LdSIR2RP2* gene in *L*. *donovani*. A fusion PCR-based strategy was employed as reported earlier [29]. Briefly, *LdSIR2RP2* flanking regions were amplified from *Ld*Bob genomic DNA and fused to antibiotic resistance cassettes: hygromycin phosphotransferase gene (HYG) or neomycin phosphotransferase gene (NEO). The 5′UTR (907 bp) of *L*. *donovani LdSIR2RP2* was amplified with primers A & B_Hyg_ or primers A & B_Neo_ (Table 1). The NEO gene (795 bp) was amplified from pX63-NEO with primers C_Neo_ & D_Neo_. The HYG gene (1012 bp) was amplified from pX63HYG with primers C_Hyg_ & D_Hyg_ (Table 1). The 3′UTR (967 bp) of *L*. *donovani LdSIR2RP2* was PCR amplified from wild-type *Ld*Bob genomic DNA using primers E_Hyg_ or E_Neo_ & antisense primer F (Table 1). The 5′UTR of *L*. *donovani LdSIR2RP2* was then ligated to the antibiotic resistance marker genes by PCR using primers A & D_Hyg_ or A & D_Neo_. Finally, this fragment (5′UTR-marker gene) was fused with 3′UTR of *LdSIR2RP2* using primers A & F, yielding the fragment, 5′UTR-Hyg-3′UTR or 5′UTR-Neo-3′UTR.

**Table 1:** Primers used for generation of the Hyg and Neo specific linear replacement cassette fragments.

S.No		*L*.*donovani* primers	Primer Sequences
**1.**	A		5’ TGATGGAGTTGCCAATCCAATTACA 3'
**2.**	B_HYG_		5’ GGTGAGTTCAGGCTTTTTCATGAGAAGATAGCAGCTGAAT 3’
**3.**	C_HYG_		5’ ATTCAGCTGCTATCTTCTCATGAAAAAGCCTGAACTCACC 3’
**4.**	D_HYG_		5’ACGCCAAGTCAAAAAGCGATCCTATTCCTTTGCCCTCGGACGAG 3’
**5.**	E_HYG_		5’CTCGTCCGAGGGCAAAGGAATAGGATCGCTTTTTGACTTGGCGT 3’
**6.**	B_NEO_		5’CAATCCATCTTGTTCAATCATGAGAAGATAGCAGCTGAAT 3’
**7.**	C_NEO_		5’ ATTCAGCTGCTATCTTCTCATGATTGAACAAGATGGATT 3’
**8.**	D_NEO_		5’ ACGCCAAGTCAAAAAGCGATCTCAGAAGAACTCGTCAAGAAG 3’
**9.**	E_NEO_		5’ CTTCTTGACGAGTTCTTCTGAGATCGCTTTTTGACTTGGCGT 3’
**10.**	F		5’ CATGAAGCTAAGAAGCAGAAA 3’

To generate the episomal ‘add back’ construct, the full-length *LdSIR2RP2* coding sequence was amplified with primers; Forward: 5′ CCCTCTAGAATGAGGCCGGCGGGGACGATC 3′ and Reverse: 5′CCCAAGCTTCTAGAGTTGAATCGTCTTGCGGCGGAAG 3′. This amplified product was then cloned into the *Xba*1 and *Hind*III restriction sites of the pSP72α-zeo-α vector to get pSP72α-zeo-α-*LdSIR2RP2* complementation construct. All the fragments and constructs were sequenced for confirmation of their correct orientation and sequence fidelity.

### Generation of genetically manipulated parasites

5′UTR-Hyg- 3′UTR or 5′UTR-Neo-3′UTR linear fragments were generated through PCR amplification. The fragments were gel purified, and about 1–2 micrograms of each fragment were individually transfected by electroporation in wild-type *L*. *donovani* promastigotes according to the standard protocol [28] Depending on the marker gene, transfectants were selected either in the presence of 200 μg/ml hygromycin (Sigma-Aldrich, USA) or 300 μg/ml paromomycin (Sigma-Aldrich, USA). The cells resistant to antibiotic selection were checked by PCR-based analysis for the correct integration of the replacement cassettes using primers shown in (Table 2). Thereafter, the second round of transfection was done to knock-out the other copy of *LdSIR2RP2* gene. The genotypes of the *LdSIR2RP2* mutants were confirmed by Southern blotting analysis using standard protocols [30].

**Table 2:** Primers used for the molecular characterization of the genetically manipulated parasites by PCR-based analysis.

S.No	*L*. *donovani* Primers	Sequences
**1.**	Primer 1	5’ TGTAGAAGTACTCGCCGATAGTGG 3'
**2.**	Primer 2	5’ TTGCTTGCCTTTCAGGAAGGGGAGGTT 3’
**3.**	Primer 3	5’ CGCAGCTATTTACCCGCAGGACAT 3’
**4.**	Primer 4	5’ CCGACTTGTTGTCTTTTCTCGTCTCC 3’
**5.**	Primer 5	5’ ATAGCGTTGG CTACCCGTGATATTGC 3’
**6.**	Primer 6	5’ AACACGGCGGCATCAGAGCAGCCGATTG 3’
**7.**	Primer 7	5’ AAACTCAATGTGGAGAGCGTCGGAA 3’
**8.**	Primer 8	5’ CAGCGCTCCATGAACGATGCGATCGT 3’
**9.**	Zeo F	5’ ATGGCCAAGTTGACCAGTGCCGTTCC 3’
**10**	Zeo R	5’ TCAGTCCTGCTCCTCGGCCACGAA 3’

The ‘add-back’ line Δ*LdSIR2RP2*/+ was created from the Δ*LdSIR2RP2* null mutants by transfecting these parasites with pSP72α-zeo-α-*LdSIR2RP2* episome. After transfection, these parasites Δ*LdSIR2RP2*/+ were selected in 800 μg/ml zeocin, 200 μg/ml hygromycin and 300 μg/ml paromomycin. Further, the genotype of the ‘add-back’ line Δ*LdSIR2RP2*/+ was confirmed by PCR analysis using primers mentioned in (Table 2).

### Growth curve analysis

Growth rate experiments were conducted by inoculating stationary-phase parasites at a density of 1 × 10^6^ cells/ml in standard M199 medium with 5% FBS in 25 cm^2^ flasks without particular selection drug and culturing at 22°C. The growth rate of each of the cultures was determined at 24 h intervals by using a Neubauer hemocytometer. Growth studies with each individual cell line were performed at least three times, and similar results were obtained consistently.

### Infectivity assay

J774A.1 murine macrophage cell line was plated on poly-L-lysine-coated glass coverslips at a density of 5 × 10^5^ cells per well in a 6-well flat bottom plate. The adherent cells were infected with stationary-phase promastigotes, at a ratio of 20:1 for 6 h. Excess non-adherent promastigotes were removed by incubation of the cells for 30 s in 1X phosphate buffer saline (1 X PBS). These were subsequently maintained in RPMI1640 containing 10% FBS at 37°C with 5% CO_2_. Intracellular parasite load was visualised by Giemsa staining.

### Cell cycle analysis

2 x 10^7^ log phase promastigotes of WT, Δ*LdSIR2RP2* and Δ*LdSIR2RP2*/+ were collected, washed twice with 1X PBS and then fixed in ice-cold 30% PBS/70% (v/v) methanol for 1 h at 4°C. The fixed cells were washed twice with ice-cold 1X PBS and then resuspended in 1 ml 1X PBS containing 100 μg/ml RNase and 20 μg/ml propidium iodide (Sigma-Aldrich, USA). The cells were incubated for 45 min at 37°C in the dark. The samples were then analysed using BD Biosciences FACS Calibur system using BD Biosciences CellQuest software. For each sample, data for at least 20,000 events were collected. The resulting distribution of cells was analysed by the Modfit Lt. Software to determine the percentage of cells in G0/G1, S, or G2/M phases of the cell cycle.

### Measurement of mitochondrial transmembrane potential (ΔΨm)

The mitochondrial transmembrane potential was investigated using MitoTracker Red CMXRos (Invitrogen). Logarithmically growing promastigotes (1 × 10^6^ cells) were incubated with Mito Tracker Red CMXRos (100 nM) for 30 minutes. Wild-type cells treated with protonophore carbonyl cyanide m-chlorophenyl hydrazone (CCCP) (50 μM) (Sigma-Aldrich, USA), a mitochondrial membrane depolarization compound was used as a control. Subsequently, the cells were washed with 1 X PBS and fixed as mentioned in the above protocol. The samples were then analysed using FACS, BD Biosciences FACS Calibur system using BD Biosciences CellQuest software. The mean fluorescence intensities (MFI) of FL2 channel were used for analysis. For each sample, data for at least 20,000 events were collected.

### Measurement of intracellular ATP levels

The cellular ATP levels of the parasites were measured using a bioluminescence-based ATP detection assay kit(BioVision) as per the manufacturer’s protocol. Briefly, 1 × 10^7^ log phase WT, Δ*LdSIR2RP2* and Δ*LdSIR2RP2*/+ cells were seeded in a 96-well plate and treated with indicated compounds (5 mM, 2-deoxyD-glucose (2DG) (Sigma-Aldrich, USA) or 10 μM Oligomycin (Oligo) (Sigma-Aldrich, USA). The cells were lysed, substrate solution was added, and the luminescence intensity was measured in a luminometer (Turners Design, TD 20/20). Percentage cellular ATP levels were plotted in reference to control.

### Inhibitor studies

The susceptibility profile of *L*. *donovani* wild-type and mutant promastigotes for sirtinol, nicotinamide (NAM), Ex-527, and cambinol was determined using MTT [3-(4, 5- dimethylthiazol-2-yl) -2, 5 diphenyltetrazolium bromide] assay [31]. Briefly, log-phase promastigotes (5 × 10^5^ cells/well) were seeded in a 96-well flat-bottomed plate and incubated with different drug concentrations at 22°C. DMSO was used as a vehicle control for inhibitors which were dissolved in DMSO. After 72 h of incubation, 20 μL of MTT (Sigma-Aldrich, USA) (5 mg/ml) was added to each well, and the plates were further incubated at 37°C for 3 h. The reaction was terminated by the addition of 50 μL of stopping solution (50% isopropanol and 20% SDS) followed by gentle shaking at 37°C for 30 min to 1 h. The absorbance was measured at 570 nm in a microplate reader (SpectraMax M2 from Molecular Devices).

The susceptibility of wild-type and mutant amastigotes to the above-mentioned inhibitors was determined by visualisation of intracellular parasite load using Giemsa staining of the infected J774A.1 murine macrophages, 48 h after treatment with different concentrations of the drug.

The cytotoxicity of inhibitors on J774A.1 murine macrophage cell line was determined by MTT assay. Briefly, 1 x 10^4^ cells per well were seeded in a 96-well flat-bottomed plate and incubated with different drug concentrations at 37°C, 5% CO_2_. DMSO was used as a vehicle control for inhibitors which were dissolved in DMSO. After 48 h of incubation, the assay was terminated by adding MTT as mentioned above and the absorbance was measured at 570 nm.

The effect of inhibitors on the ADP-ribosyltransferase activity of recombinant *Ld*SIR2RP2 was assessed. Briefly, the assays were carried out with 2.5 μg of recombinant *Ld*SIR2RP2, 5 μg of calf thymus histones (Sigma), and the respective inhibitors at indicated concentrations. The reaction mixtures were incubated at 37°C for 1 h. Thereafter, 2.5 μCi of [^32^P]NAD^+^ was added to the above mixture, and the reactions were allowed to proceed further at room temperature for 2 h. The reactions were terminated, resolved on 12% SDS gel and visualized as stated above.

### Statistical analysis

Statistical analysis was done using Graph Pad Prism Version 5.0. Data shown are representative of at least three independent experiments unless otherwise stated as n values given in the legend. All the experiments were set in triplicate, and the results are expressed as the mean ± S.D. Student’s t test was employed to assess the statistical significance of differences between a pair of data sets with a *p-value* of < 0.05 considered to be significant.

## Results

### Sequence analysis and phylogeny

**Fig 1:**
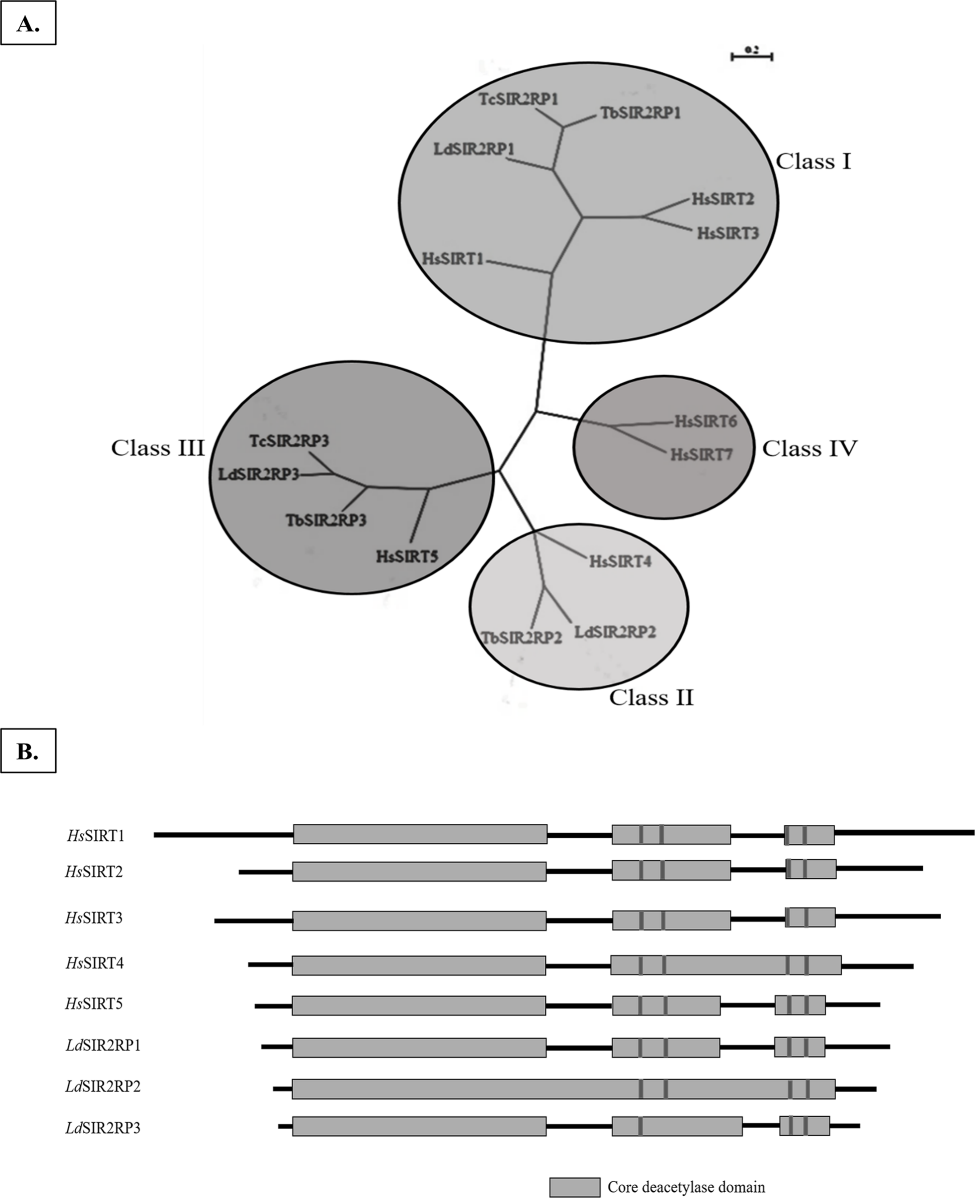
Phylogenetic analysis of *Leishmania* sirtuins. (A) The unrooted neighbor-joining tree was generated using MUSCLE program and Unrooted software. The sequences are divided into four sirtuin classes; I, II, III and IV based on the conserved sirtuins core deacetylase domain. (B). Schematic representation of the predicted *L*. *donovani* sirtuins compared with *Hs*SIRT1, *Hs*SIRT2, *Hs*SIRT3, *Hs*SIRT4, and *Hs*SIRT5. The location of Cys residues that form the Zn^2+^ -binding motif are indicated (black bars).

The eukaryotic sirtuins are classified into four classes: I, II, III and IV, based on the conserved sequence motif patterns [7]. Humans have seven sirtuins distributed in all the four classes [32] while kinetoplastids have Class I, Class II, and Class III sirtuins (Fig 1A). There are three SIR2 related proteins in *L*. *donovani*. The SIR2 related proteins of *L*. *donovani* (*Ld*BPK_260200.1, *Ld*BPK_231450.1, and *Ld*BPK_341900.1) are termed as *Ld*SIR2RP1, *Ld*SIR2RP2, and *Ld*SIR2RP3. The phylogenetic analysis of kinetoplastid sequences along with the homologs from human (Fig 1A) suggests a clear branching of the individual sirtuin subfamilies. *Ld*SIR2RP1 belongs to Class I; *Ld*SIR2RP2 belongs to Class II, and *Ld*SIR2RP3 belongs to Class III, of sirtuins. Class I, kinetoplastid sequences are related to *Hs*SIRT1, *Hs*SIRT2, and *Hs*SIRT3. Class II kinetoplastid sequences are closer to *Hs*SIRT4. Further, it is also evident from the phylogeny that class III kinetoplastid sirtuins are closely related to *Hs*SIRT5 sirtuin that have been shown to possess both NAD^+^-dependent deacetylase and the novel desuccinylase/ demalonylase activities [12]. To date, the Class I homologs of *Leishmania* [15, 21] and *Trypanosoma [13, 17]* parasites have been characterized, and nothing is known about the sirtuins belonging to Class III and Class II subfamilies. The *Ld*SIR2RP1, *Ld*SIR2RP2, and *Ld*SIR2RP3 encode putative polypeptides of amino acids 373, 320 and 243, respectively. The predicted molecular mass of *Ld*SIR2RP1, *Ld*SIR2RP2 and *Ld*SIR2RP3 is 41, 35 and 27 kDa, respectively. Of these, the SIR2 *Leishmania* homolog *Ld*BPK_260200.1 was predicted to be localized in the cytosol. The other two SIR2 copies on Chromosome 34 (*Ld*BPK_341900.1) and Chromosome 23 (*Ld*BPK_231450.1) are predicted to be localized in the mitochondria. All the three *Leishmania* sirtuins lack the N-terminal extension present in *Hs*SIRT1 and *Hs*SIRT2 that are required for nucleolar localization [33] but contain a full catalytic SIR2 domain (Fig 1B). These proteins also have the conserved Zn^2+^ binding motif (CX_2_CX_20_CX_2_C), although one of the Cys residues is lacking in *Ld*SIR2RP3 (Fig 1B).

**Fig 2:**
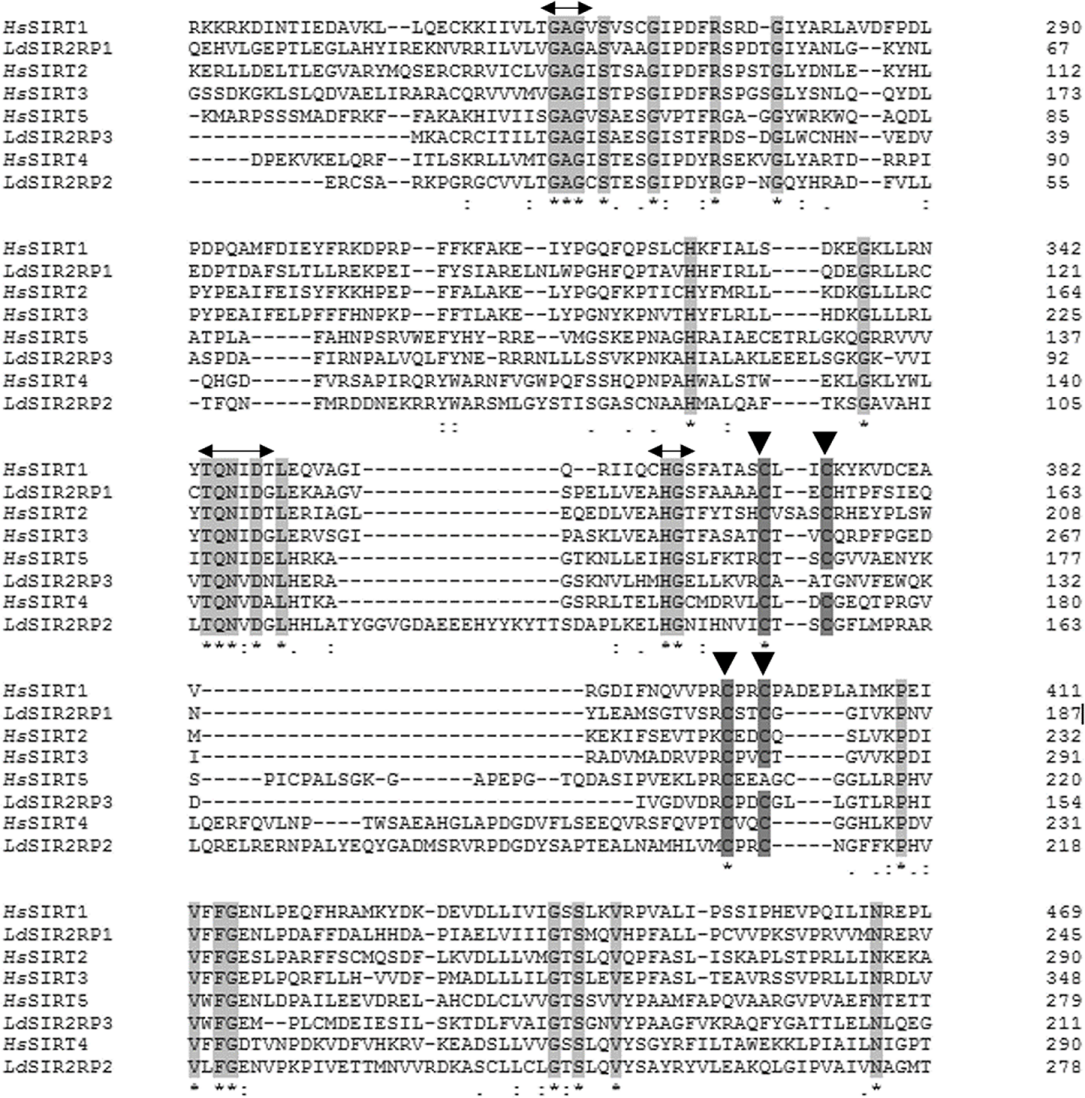
Sequence analysis. Multiple sequence alignment of *Leishmania* sirtuins with *Hs*SIRT1, *Hs*SIRT2, *Hs*SIRT3, *Hs*SIRT4 and *Hs*SIRT5 using Clustal Omega. The conserved GAG, TQNID, and HG motifs are highlighted in light grey, The Cys residues from the Zn^2+^-binding domain are shown in dark grey (arrowheads).

Multiple sequence alignment of the catalytic region of *Leishmania* sirtuins with that of human sirtuins homologs shows the conserved sequence patterns characteristic of the individual subfamilies of sirtuins (Fig 2). GAG, TQNID and HG motifs, as well as other residues essential for enzymatic catalysis, are conserved in *Leishmania* sirtuins. Among the conserved motifs, the “HG” motif is of interest as mutation of “HG” to “YG” has been shown to convert the yeast sirtuin into a dominant negative gene with loss in function [7] while it is conserved as “QG” in microbial sirtuins denoted as SirTM subfamily [34]. Thus, the “HG” motif essential for sirtuin-mediated ADP-ribosylation and deacetylation is conserved in all the kinetoplastid sequences suggestive of active sirtuins. Both *Ld*SIR2RP1 and *Ld*SIR2RP2 contain a zinc binding motif. Overall, *Ld*SIR2RP1 sequence shares 46% sequence identity with its human homolog *Hs*SIRT2, while *Ld*SIR2RP2 and *Ld*SIR2RP3 share 39% and 37% sequence identity with *Hs*SIRT4 and *Hs*SIRT5, respectively.

### *Ld*SIR2RP2 of *L*. *donovani* is an NAD^+^- dependent ADP-ribosyltransferase

**Fig 3:**
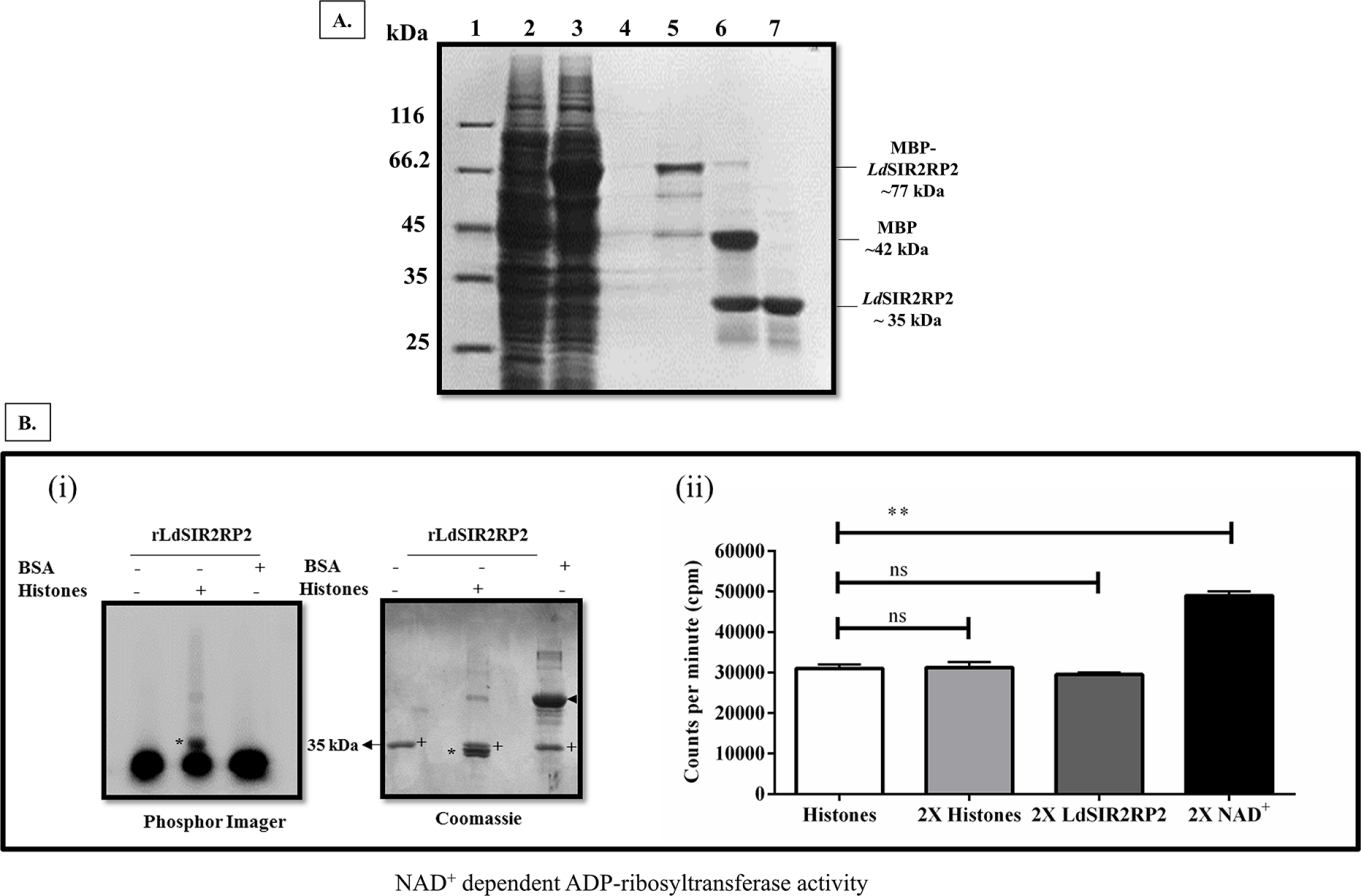
Expression, purification and enzymatic characterization of *Ld*SIR2RP2. (A). Lane 1, molecular weight marker; Lane 2, uninduced cell lysate; Lane 3, induced cell lysate; Lane 5, concentrated eluates showing purified protein; Lane 6, Purified protein after TEV protease cleavage; Lane 7, *Ld*SIR2RP2 protein without the MBP tag after passing through second amylose column. (B)(i). *In vitro* ribosylation reaction of r*Ld*SIR2RP2 (**+**), was performed using [^32^P] NAD^+^ as donor and BSA or calf thymus histones as acceptor protein. The reaction products were resolved by 12% SDS-PAGE and analyzed by PhosphorImager and Coomassie blue stain. (B)(ii). Quantitative analysis of the effect of NAD^+^, histones, and r*Ld*SIR2RP2 on the extent of ribosylation by r*Ld*SIR2RP2. Ribosyltransferase reactions were performed with 5 μg of histones, 2.5 μg of r*Ld*SIR2RP2 and 2.5 μCi of [^32^P]NAD^+^ (standard reaction) or a double concentration of histone (10 μg, 2X histones), r*Ld*SIR2RP2 (5 μg, 2X r*Ld*SIR2RP2) and [^32^P]NAD^+^ (5 μCi, 2X NAD^+^). The reaction products were precipitated with 20% TCA, collected, washed and then counted after the addition of scintillation liquid.

In order to overexpress the recombinant *Ld*SIR2RP2, the coding sequence of *LdSIR2RP2* was cloned into a pETM41 expression vector possessing an N-terminal maltose binding protein (MBP) tag. The construct was transformed into Artic-Express DE3 strain and induced as explained in the Methods section, resulting in expression of MBP-tagged recombinant *Ld*SIR2RP2 with an estimated molecular size of ~ 77 kDa (Fig 3A). The size of the recombinant protein correlated with the amino acid composition of the *Ld*SIR2RP2 protein (~ 35 kDa) and MBP tag (~ 42 kDa). The recombinant MBP-*Ld*SIR2RP2 was affinity purified on a pre-equilibrated amylose resin column yielding ∼1 mg of pure protein from 1 litre of bacterial culture. The MBP tag cleavage of the recombinant protein was done by using TEV protease at a w/w ratio of 1% the amount of fusion protein at 4°C. The pure recombinant *Ld*SIR2RP2 was obtained after passing the reaction mixture, first through pre-equilibrated Ni^2+^-NTA column to remove TEV protease and then through pre-equilibrated amylose resin column to remove the MBP tag protein (Fig 3A).

Proteins belonging to the SIR2 family exhibit NAD^+^-dependent deacetylase activity due to the presence of a well-conserved enzymatic core SIR domain of ∼250 amino acids [7, 35]. Some members of the SIR2 family are also known to catalyze the transfer of ribose 5′-phosphate from nicotinic acid mononucleotide to amino acid residues of bovine serum albumin (BSA), histones or SIR2 proteins themselves [11, 35]. Recently, class III sirtuins have been reported to have novel enzymatic activities like desuccinylase and demalonylase [12, 36]. Using recombinant *Ld*SIR2RP2, we checked NAD^+^-dependent deacetylase and/or NAD^+^-dependent ADP-ribosyltransferase activity and/or desuccinylase activities. However, the recombinant protein did not show any detectable deacetylase and desuccinylase activities.

Next, to assess whether *Ld*SIR2RP2 is an ADP-ribosyltransferase, [^32^P]NAD^+^ as the donor and bovine serum albumin (BSA) or calf thymus histone as acceptor substrates were used. r*Ld*SIR2RP2 protein was able to catalyze the ADP-ribosylation of calf thymus histones (Fig 3B). However, there was no transfer of ADP-ribose on BSA, suggesting its strong specificity towards histones. Quantitative analysis of r*Ld*SIR2RP2 done using histones as acceptor protein showed that a two-fold increase in the number of histones and recombinant protein did not have any effect on the extent of ADP-ribosylation. However, when the amount of NAD^+^ was doubled, there was ~1.5-fold increase in ADP-ribosylation (Fig 3B(ii)), suggesting that NAD^+^ is the only limiting factor for ADP-ribosylation activity of r*Ld*SIR2RP2. Phylogenetically *Ld*SIR2RP2 belongs to class II of sirtuin family, of which *Hs*SIRT4 is a prominent member. *Hs*SIRT4 only has NAD^+^-dependent ADP-ribosyltransferase activity [37]. Thus, it can be concluded that like its human counterpart, *Ld*SIR2RP2 possessed an NAD^+^-dependent ADP-ribosyltransferase activity and lacked measurable NAD^+^-dependent deacetylase and desuccinylase activities. However, this needs to be further validated using stringent purification methods.

### *LdSIR2RP2* is localized to the mitochondria

**Fig 4:**
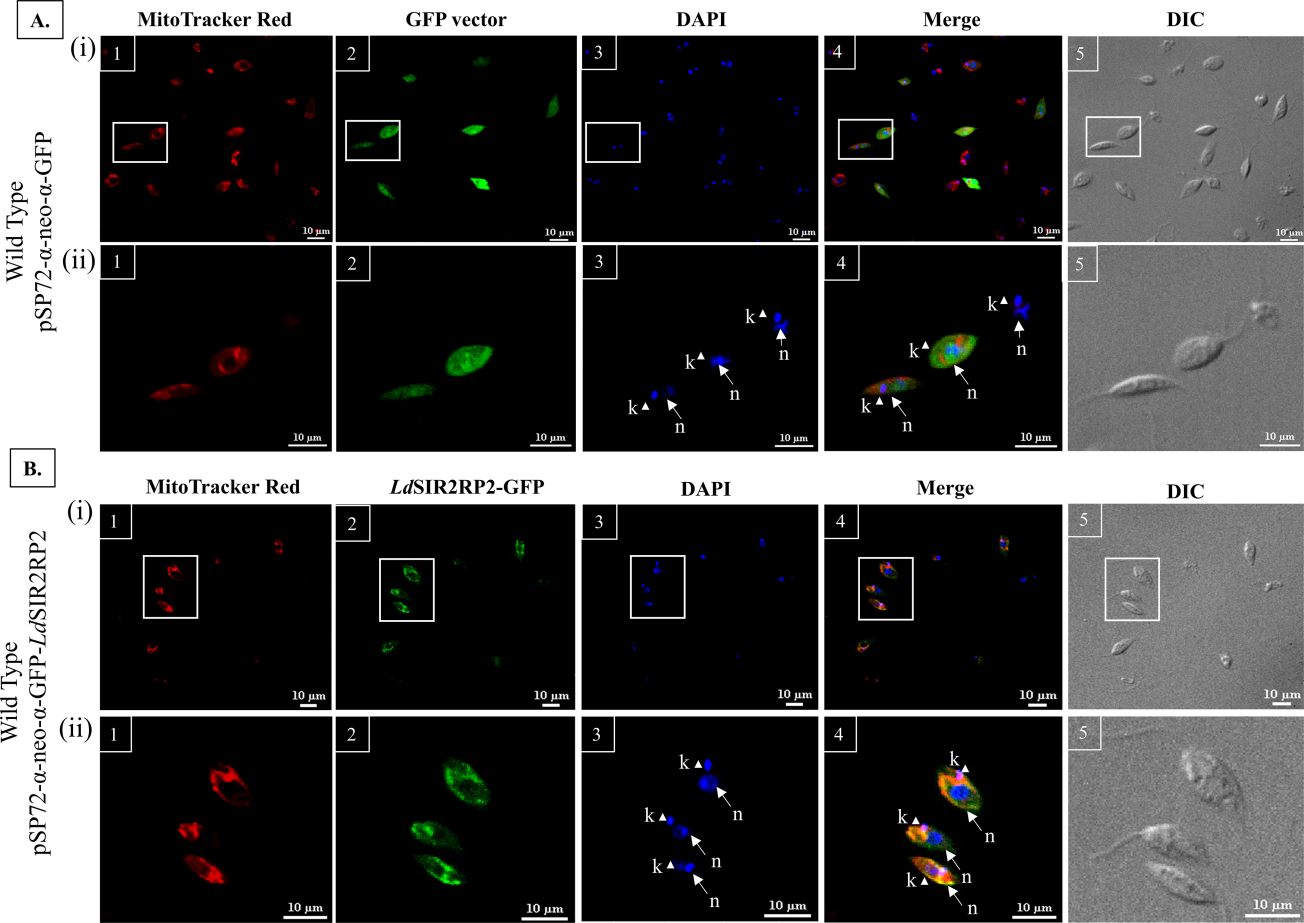
Cellular distribution of *Ld*SIR2RP2. (A) Confocal microscopy of wild-type *L*. *donovani* transfected with pSP72-α-neo-α-GFP. (i) Large field vision at 60x (ii) Magnifications of the boxed areas in the top images. Panel 1, wild-type *L*. *donovani* stained with MitoTracker Red; Panel 2, wild-type *L*. *donovani* expressing GFP protein; Panel 3, wild-type *L*. *donovani* stained with DAPI; Panel 4, Merged micrograph; Panel 5, Phase contrast micrograph. (B) Confocal microscopy of wild-type *L*. *donovani* transfected with pSP72-α-neo-α-*LdSIR2RP2*-GFP. (i) Large field vision at 60x (ii) Magnifications of the boxed areas in the top images. Panel 1, wild-type *L*. *donovani* stained with MitoTracker Red; Panel 2, wild-type *L*. *donovani* expressing *Ld*SIR2RP2-GFP fusion protein; Panel 3, wild-type *L*. *donovani* stained with DAPI; Panel 4, Merged micrograph; Panel 5, Phase contrast micrograph. n, nuclear DNA; k, kinetoplast DNA. Scale bar represents 10 μm.

*In-silico* analysis performed with PSORTII software indicated a higher probability for a mitochondrial localisation of *Ld*SIR2RP2. The subcellular localisation of *Ld*SIR2RP2 was confirmed using confocal microscopy to evaluate the localisation of C-terminal GFP-tagged full-length *Ld*SIR2RP2. *L*. *donovani* parasites transfected with the *LdSIR2RP2*-GFP fusion constructs and parasites transfected with GFP vector alone (without insert) were fixed and analysed by fluorescence microscopy. The GFP fluorescence was visible in approximately 80% of the cells (Fig 4A (i)). The kinetoplast and nuclear DNA in these cells were readily identified by their bright staining with DAPI. The parasites transfected with the GFP vector alone showed GFP fluorescence in the entire promastigotes (Fig 4A). However, the *Ld*SIR2RP2-GFP fusion protein was found to localise in the mitochondria as seen by the co-localization of the *Ld*SIR2RP2−GFP with the fluorescence associated to the MitoTracker Red CMXRos that reveals the position of mitochondria within the promastigotes (Fig 4B).

### Gene deletion studies of *Ld*SIR2RP2

In order to determine the essentiality and biological function(s) of the mitochondrial sirtuin in *L*. *donovani*, we replaced both the alleles of *Ld*SIR2RP2 gene using classical gene replacement experiments.

Two successive rounds of gene targeting with two dominant selectable markers were undertaken to inactivate the *LdSIR2RP2* gene completely. This was done by the generation of inactivation cassettes having hygromycin phosphotransferase (*HYG*) or neomycin phosphotransferase (*NEO*) as selection markers along with 5′UTR and 3′UTR of *LdSIR2RP2* gene, as described in the Methods. Linear replacement cassette fragments were transfected into wild-type *L*. *donovani* promastigotes leading to the generation of heterozygous parasites in which one copy of *LdSIR2RP2* gene was replaced with either the hygromycin or neomycin drug resistance gene.

**Fig 5:**
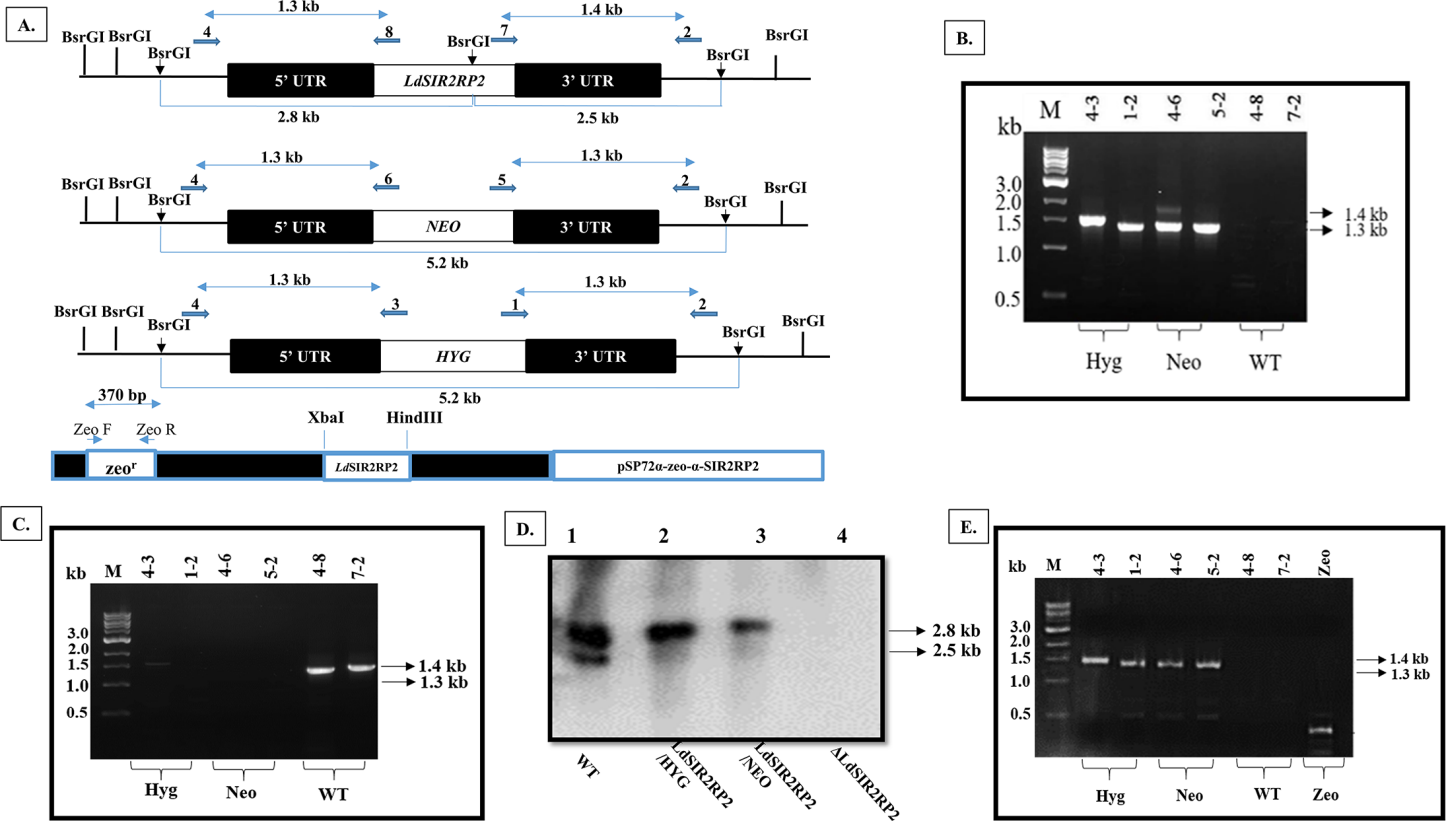
Generation of deletion mutants of *LdSIR2RP2* in *L*. *donovani*. (A) Restriction map of the *LdSIR2RP2* locus and pSP72α-zeo-α-*LdSIR2RP2* along with the location of the primers used for confirmation by PCR analysis. *Bsr*GI restriction enzyme was used to analyse the recombination event. Primer 4 was designed as a forward primer to match the upstream region of *LdSIR2RP2* gene, and primers 8, 3 and 6 were designed internally to *LdSIR2RP2*, *HYG*, and *NEO* coding regions, respectively. Primer 2 was designed as a reverse primer to match the downstream region of *LdSIR2RP2* gene, and primers 7, 1 and 5 were designed as forward primers, internal to *LdSIR2RP2*, *HYG*, and *NEO* coding regions, respectively. Primers ZeoF and ZeoR were designed as forward and reverse primers for *Sh ble* gene that codes for Zeocin antibiotic resistance. (B) Genomic DNA from Δ*LdSIR2RP2* parasites was used as a template for PCR analysis. The specificity of recombination event was checked with *HYG*, *NEO* and *LdSIR2RP2* (WT) gene specific primers. M indicates the DNA molecular size marker in kb. (C) Genomic DNA from WT parasites was used as a template for PCR analysis with *HYG*, *NEO* and *LdSIR2RP2* (WT) gene specific primers. M indicates the DNA molecular size marker in kb. (D) Southern blot analysis of wild-type *Bob* (WT), heterozygotes *LdSIR2RP2 /NEO*, *LdSIR2RP2/HYG* and Δ*LdSIR2RP2* null mutant parasites. An equal amount of genomic DNA was digested with *Bsr*GI, resolved on a 0.6% agarose gel and hybridised with a *LdSIR2RP2* gene specific probe. Molecular weight marker is indicated to the right of the blot. (E) PCR analysis of Δ*LdSIR2RP2* parasites transfected with pSP72α-zeo-α-*LdSIR2RP2* (Δ*LdSIR2RP2/+*). The specificity of recombination event was checked with *HYG*, *NEO*, *LdSIR2RP2* (WT) and *Sh ble* (Zeo^R^) gene specific primers. M indicates the DNA molecular size marker.

Subsequently, another round of gene targeting was done to generate *LdSIR2RP2* homozygous null mutant parasites. PCR analysis was done to confirm the recombination events (Fig 5A and 5B). Genomic DNA from the WT parasites was used as a positive control (Fig 5C). Bands corresponding only to the *LdSIR2RP2* gene were obtained indicating the specificity of *HYG* and *NEO* primers. The genotype of the heterozygous (*LdSIR2RP2*/*Hyg* and *LdSIR2RP2*/*Neo*) and homozygous (Δ*Ld*SIR2RP2) null mutant parasites was further confirmed by Southern blot analysis (Fig 5D).

The ‘add-back’ mutant line (Δ*LdSIR2RP2*/+) was prepared by transfecting pSP72a-zeo-a-*LdSIR2RP2* episome into homozygous null mutant parasites (Δ*LdSIR2RP2*). PCR analysis was done to confirm the presence of the episomal plasmid (Fig 5A and 5E). A band of ~ 360 bp was obtained upon amplification with ZeoF and ZeoR primers, which correspond to *Sh ble* gene, that confers Zeocin antibiotic resistance [38], thus confirming the presence of episomal pSP72a-zeo-a-*LdSIR2RP2* in the ‘add-back’ line (Δ*LdSIR2RP2*/+).

### *LdSIR2RP2* is essential for growth as well as infectivity

**Fig 6:**
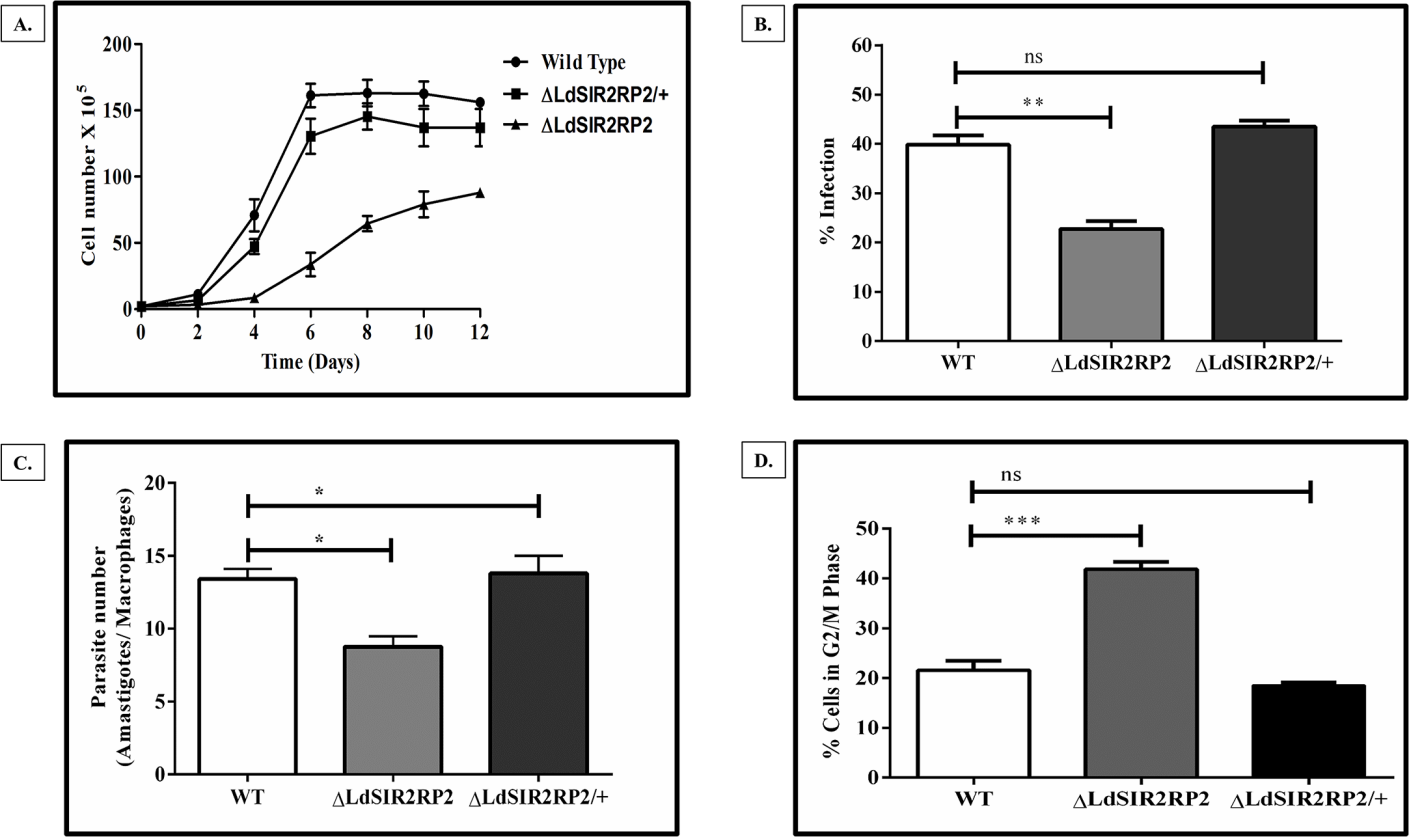
Phenotypic characterisation of Δ*LdSIR2RP2* null mutant parasites. (A) Growth curve analysis of *L*. *donovani* wild-type (WT), *LdSIR2RP2* homozygous (Δ*LdSIR2RP2*) null mutants and *LdSIR2RP2* ‘add-back’ mutant (Δ*LdSIR2RP2/+*). The experiment was repeated thrice independently in triplicate. The representative data from one experiment is shown. (B) and (C): Comparison of infectivity (B) and parasite load (C) of *L*. *donovani* wild-type (WT), null mutant (Δ*LdSIR2RP2*) and ‘add-back’ mutant (Δ*LdSIR2RP2/+*) parasites in J774A.1 murine macrophage cell line. Murine macrophage cell line J774A.1 was infected with stationary-phase promastigotes at an MOI of 20:1. Cells were stained with Giemsa after 48 h and amastigotes were enumerated visually. The results represent mean ± SD with n = 3. * *p* < 0.05; **, *p* < 0.01, *** *p* < 0.005 and ns indicates not significant (*p* > 0.05). (D) Bar graph depicting the percentage of cells in the G2/M phase of WT, Δ*LdSIR2RP2 and* Δ*LdSIR2RP2/+*. The data represents mean ± SD with *n* = 3. * *p* < 0.05; **, *p* < 0.01, *** *p* < 0.005 and ns indicates not significant (*p* > 0.05).

The growth rate of each of the cell line was determined in order to verify phenotypic alterations in the wild-type and genetically manipulated parasites. This was done by counting promastigote cells using a hemocytometer for a period of 12 days. The absence of *Ld*SIR2RP2 in the promastigotes led to a significant decrease in the growth rate of the parasite as compared to the WT cells (Fig 6A). The doubling time of the Δ*LdSIR2RP2* (~ 32 h) was ~ 2.5 fold higher than the WT cells (~ 12 h). This restrictive growth phenotype was rescued in the promastigotes expressing episomal *LdSIR2RP2* (Δ*LdSIR2RP2*/+) with doubling time of ~ 14 h.

Next, we assessed whether the genetic deficiency of *LdSIR2RP2* in *L*. *donovani* has an impact on its ability to infect host cells by performing infectivity assays with stationary-phase promastigotes in the J774A.1 murine macrophage. Microscopic observation of murine macrophages stained with Giemsa showed that, while WT parasites were capable of ~ 50% infection in murine macrophages, Δ*LdSIR2RP2* parasites had reduced infectivity with only ~ 25% of the macrophages infected (Fig 6B). The add-back line (Δ*LdSIR2RP2*/+) showed infection comparable to that of WT (Fig 6B). Upon comparing the parasite numbers of the WT, Δ*LdSIR2RP2*, and Δ*LdSIR2RP2*/+ in the murine macrophages, it was observed that Δ*LdSIR2RP2* mutant parasites had ~ 50% reduction in the number of amastigotes per macrophages relative to the wild-type parasites 48 h p.i (Fig 6C). In the ‘add-back’ line (Δ*LdSIR2RP2*/+) the parasite numbers were restored to the levels comparable to that of WT. Thus, these observations imply that loss of *Ld*SIR2RP2 affects the ability of the parasite to infect and sustain robust infection within murine macrophages. This could be partially attributed to the slow growth phenotype of the null mutants compared to their wild-type counterparts.

Since the Δ*LdSIR2RP2* parasites exhibited a restrictive growth phenotype, the possibility of any cell cycle-related defects, which could have lowered the growth rate of mutant parasites, was examined. For this, log phase cells of WT, Δ*LdSIR2RP2*, and Δ*LdSIR2RP2*/+ parasites were taken and examined for their DNA content. The null mutants showed an increased G2/M population of cells (~ 40.33%) (*p* = 0.0001) compared to the WT (23.68%) and ‘add-back lines (17.79%) (Fig 6D). It is possible that the G2/M block in the null mutants may be indirectly responsible for the slow growth kinetics of Δ*LdSIR2RP2* parasites.

### *LdSIR2RP2* is required for mitochondrial function

**Fig 7:**
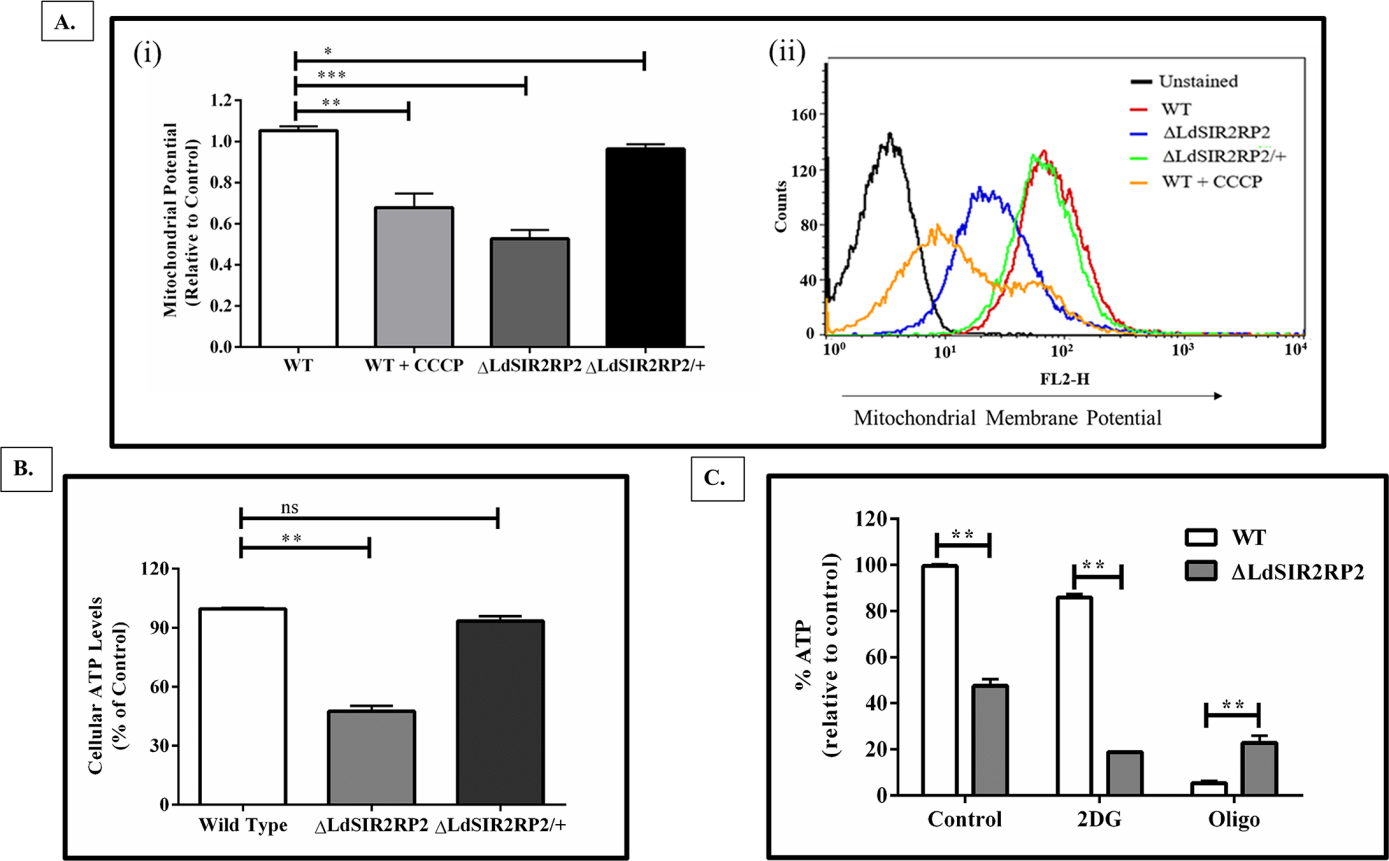
Deletion of *Ld*SIR2RP2 affects mitochondrial functioning in parasites. (A)(i) The mitochondrial membrane potential of WT, Δ*LdSIR2RP2*, and Δ*LdSIR2RP2*/+ cells was monitored using the fluorescent probe MitoTracker Red. Cells were incubated with 100 nM of MitoTracker Red, washed, fixed and analysed by BD Biosciences FACS Calibur system using BD Biosciences CellQuest software. Uncoupling agent CCCP (50 μM) was used as a positive control. Percent Mean Fluorescence Intensity (MFI), relative to WT control was plotted. (ii) Histograms showing an overlay of mitochondrial membrane potential of WT, WT treated with CCCP, Δ*LdSIR2RP2*, and Δ*LdSIR2RP2*/+ cells. The x-axis shows the log FL-2 channel fluorescence intensity and the y-axis indicate the cell number. (B) Total cellular ATP levels of WT, Δ*LdSIR2RP2*, and Δ*LdSIR2RP2*/+ cells were determined using ATP/ADP determination kit (BioVision). ATP levels were calculated relative to 100% of the control WT. (C) WT and Δ*Ld*SIR2RP2 cells were incubated with either M199 medium plus 10 μM Oligomycin to inhibit mitochondrial ATP generation or M199 medium plus 5 mM 2-deoxyD-glucose (2DG), to inhibit glycolytic ATP generation. Afterwards, cells were washed, and ATP levels were determined using ATP/ADP determination kit (BioVision). ATP levels were calculated relative to 100% of the control WT. All the results represent mean ± SD with n = 3. * *p* < 0.05; **, *p* < 0.01, *** *p* < 0.005 and ns indicates not significant (*p* > 0.05).

Since *Ld*SIR2RP2 had mitochondrial localization, the effect of *LdSIR2RP2* deletion on the functioning of the parasite mitochondria was investigated. Mitochondria utilise oxidation of substrates to produce membrane potential in the form of a proton gradient across the inner mitochondrial membrane. Maintenance of this potential is necessary for the generation of ATP by mitochondria [39]. Hence, the effect of *LdSIR2RP2* gene deletion on the mitochondrial transmembrane potential (ΔΨm) was evaluated by using MitoTracker Red. MitoTracker Red is known to accumulate in energised mitochondria [40]. Relative to wild-type control, Δ*LdSIR2RP2* mutants showed decreased MitoTracker red fluorescence by ~ 48.33% thus indicating reduced ΔΨm (Fig 7A). Δ*LdSIR2RP2* mutants expressing episomal *Ld*SIR2RP2 showed ΔΨm comparable to that of the WT control. WT cells treated with 50 μM CCCP were used as positive controls. These cells showed a decrease in the mean fluorescence intensity values (32.67% of reduction) as compared to the untreated WT cells.

The alterations in the mitochondrial membrane potential are known to affect the levels of cellular ATP. Since the null mutants exhibited reduced ΔΨm, intracellular levels of ATP were determined. Δ*LdSIR2RP2* cells exhibited a significant decline (47%) in cellular ATP levels when compared to the wild-type controls (Fig 7B). The ATP levels of rescue mutants (Δ*LdSIR2RP2*/+) cell were restored to that of the WT cells. The present data indicates the effect of *LdSIR2RP2* deletion on mitochondrial membrane potential and hence, ATP synthesis in the cell.

Furthermore, we delineated whether this decline in total cellular ATP levels was due to a decrease in the mitochondrial or glycolytic ATP synthesis. For this, WT and Δ*LdSIR2RP2* parasites were treated either with oligomycin, a classical inhibitor of F0-F1-ATP synthase or 2-deoxyD-glucose (2DG), a competing substrate for hexokinase. It was observed that the WT cells treated with 2DG, exhibited higher ATP levels (85%) in comparison to Δ*LdSIR2RP2* (20%) when treated with 2DG (Fig 7C). This implied that the decline in total cellular ATP pool in Δ*LdSIR2RP2* was due to a reduction in the mitochondrial generated ATP. On the contrary, when the cells were treated with 10 μM of oligomycin, WT parasites had only 5% ATP levels as opposed to 22.5% ATP levels in Δ*LdSIR2RP2* parasites (Fig 7C), suggesting that the glycolytic ATP generation is higher in Δ*LdSIR2RP2* parasites than the WT parasites. This is in accordance with the observation that when the ability of parasites to generate ATP through mitochondrial oxidative phosphorylation is compromised, parasites increase their glycolytic metabolism to maintain the energy supply [41].

### Effect of sirtuin inhibitors on the growth of WT, Δ*LdSIR2RP2*, and Δ*LdSIR2RP2*/+ parasites

The structural and biochemical differences between the human and the parasitic sirtuins have led to exploring sirtuins as potential anti-parasitic therapeutic targets [42]. The effect of sirtuin inhibitors: sirtinol, nicotinamide (NAM), 6-chloro-2,3,4,9-tetrahydro-1H-carbazole-1-carboxamide (Ex-527) and cambinol, on the growth of WT, Δ*LdSIR2RP2*, and Δ*LdSIR2RP2*/+ parasites, was determined.

**Fig 8:**
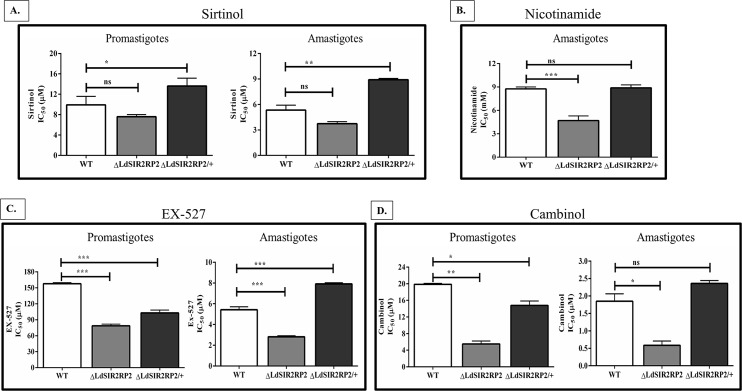
Effect of sirtuin inhibitors on the growth of WT, Δ*LdSIR2RP2*, and Δ*LdSIR2RP2*/+ parasites. The leishmanicidal effect of sirtuin inhibitors was tested on the promastigotes and the intracellular amastigotes of WT, Δ*Ld*SIR2RP2 and Δ*Ld*SIR2RP2/+ strains. The mean IC_50_ values were calculated for each inhibitor and are plotted as bar graphs. A, B, C and D: Effect of A, Sirtinol; B, Nicotinamide (NAM); C, Ex-527; and D, Cambinol; on the growth of promastigotes and intracellular amastigotes. The bar graphs represent mean ± SD with *n* = 3. * *p* < 0.05; **, *p* < 0.01, *** *p* < 0.005 and ns indicates not significant (p > 0.05).

Sirtinol is a naphthol derivative compound and is a known inhibitor of NAD^+^-dependent deacetylase activity of sirtuins. Treatment of WT, Δ*LdSIR2RP2*, and Δ*LdSIR2RP2*/+ with sirtinol inhibited the growth of both the promastigote and the amastigote stage of the parasites in a concentration-dependent manner. The IC_50_ of sirtinol for the promastigotes of WT was not significantly different from that of Δ*LdSIR2RP2* (*p* = 0.08). Similar observation was made in the case of intracellular amastigotes (*p* = 0.09) (Fig 8A, Table 3).

**Table 3:** Effect of sirtuin inhibitors on the growth of WT, Δ*LdSIR2RP2*, Δ*LdSIR2RP2/+* parasites and murine macrophages J774A.1 cell lines.

Inhibitor	Promastigotes			Amastigotes			Murine Macrophages
	Wild Type	Δ*LdSIR2RP2*	Δ*LdSIR2RP2*/+	Wild Type	Δ*LdSIR2RP2*	Δ*LdSIR2RP2*/+	J774A.1
**Sirtinol (μM)**	10.8 ± 2.5	7.6 ±1.4*	13.9 ± 3.3	5.0 ± 1.5	3.2 ± 0.13*	8.7 ± 3.16	21.6 ± 3.2
**NAM (mM)**	N.D.	N.D.	N.D.	8.8 ± 4.4	5.1 ± 2.2	9.2 ± 3.2*	29.2 ± 5.3
**EX-527 (μM)**	157 ± 3.1	80 ± 2.5	101 ± 2.9	5.7 ± 3.4	2.8 ± 0.2	7.9 ± 2.6	157 ± 4.4
**Cambinol (μM)**	19.7 ± 4.1	5.1 ± 6.8	15.5 ± 6.4	1.7 ± 0.02	0.7 ± 0.1	2.4 ± 0.2*	188 ± 11.6

Values are expressed as mean ± S.D of experiments performed in triplicates. IC_50_ values were calculated using 0.1 μM- 200 μM of sirtinol, 0.5 mM– 20 mM of nicotinamide, 0.01 μM- 400 μM of EX-527 and 0.01 μM- 300 μM of cambinol. The cytotoxicity of the inhibitors to the murine macrophages was determined using the same concentrations of the inhibitors.

*indicates non-significant *P* value compared to WT. N.D: not determined.

Nicotinamide (NAM) is a known physiological inhibitor of SIR2 deacetylase activity of *Hs*SIRT1 and *Sc*SIR2 [43]. Concentrations of NAM as high as 10 mM did not inhibit the growth of the promastigote stage of WT, Δ*LdSIR2RP2* and Δ*LdSIR2RP2*/+ parasites (Table 3). This could be attributed to the presence of thick lipophosphoglycan layer around the promastigotes that could interfere with the entry of the inhibitor inside the promastigotes. However, NAM inhibited the proliferation of intracellular amastigotes of WT, Δ*LdSIR2RP2*, and Δ*LdSIR2RP2*/+. The IC_50_ value of NAM was significantly lower in the case of Δ*LdSIR2RP2* than that of WT parasites. The IC_50_ value of NAM in the rescue mutants (Δ*LdSIR2RP2*/+) was comparable to that of the WT parasites (Fig 8B). NAM was also tested for its cytotoxicity to J774A.1 and was found to inhibit its growth at an IC_50_ value of 29.21 ± 5.3 mM, which was ~3 fold higher than that observed for the intracellular amastigotes (Table 3).

EX-527 is an indole-based sirtuin inhibitor. WT, Δ*LdSIR2RP2*, and Δ*LdSIR2RP2*/+, promastigotes were susceptible to EX-527 inhibition. The IC_50_ value of EX-527 for null mutants was ~2 fold lower than that of the WT parasites. The IC_50_ of EX-527 for Δ*LdSIR2RP2*/+ was comparable to that of the WT parasites (Fig 8C, Table 3). EX-527 was found to be a more effective inhibitor in the case of intracellular amastigotes of all parasitic cell lines (Table 3). The IC_50_ value of EX-527 for Δ*LdSIR2RP2* was ~2 fold lower than that for the WT intracellular amastigotes. EX-527 inhibited J774A.1 mouse macrophages at higher concentrations (IC_50_: 157 ± 4.4 μM) when compared to that observed in the case of the intracellular amastigotes.

Cambinol, a potent human SIRT1 and SIRT2 inhibitor [44], inhibited the growth of WT, Δ*LdSIR2RP2*, and Δ*LdSIR2RP2*/+ promastigotes (Fig 8D, Table 3). The IC_50_ value of cambinol for Δ*LdSIR2RP2* was ~4 fold lower than that of the WT promastigotes. Cambinol inhibited the growth of WT, Δ*LdSIR2RP2* and Δ*LdSIR2RP2*/+ intracellular amastigotes at the IC_50_ value of 1.7 ± 0.02 μM, 0.8 ± 0.1 μM and 2.4 ± 0.2 μM, respectively, (Fig 8D, Table 3). The compound was found to be cytotoxic to the J774A.1 mouse macrophages (IC_50_: 188 ± 11.6 μM).

Our data indicates increased susceptibility of null mutants to all the tested compounds except sirtinol. This would indicate the possible pleiotropic effect of these inhibitors on the other two parasitic sirtuins, *Ld*SIR2RP1, and *Ld*SIR2RP3. Earlier studies have also shown that NAM inhibits recombinant *Li*SIR2RP1 (15). Furthermore, overexpression of either of these sirtuins, *Tc*SIR2RP1 and *Tc*SIR2RP3 in *T*. *cruzi* protected the parasite from the effect of cambinol and NAM (17).

### Effect of sirtuin inhibitors on the activity of recombinant *Ld*SIR2RP2

**Fig 9:**
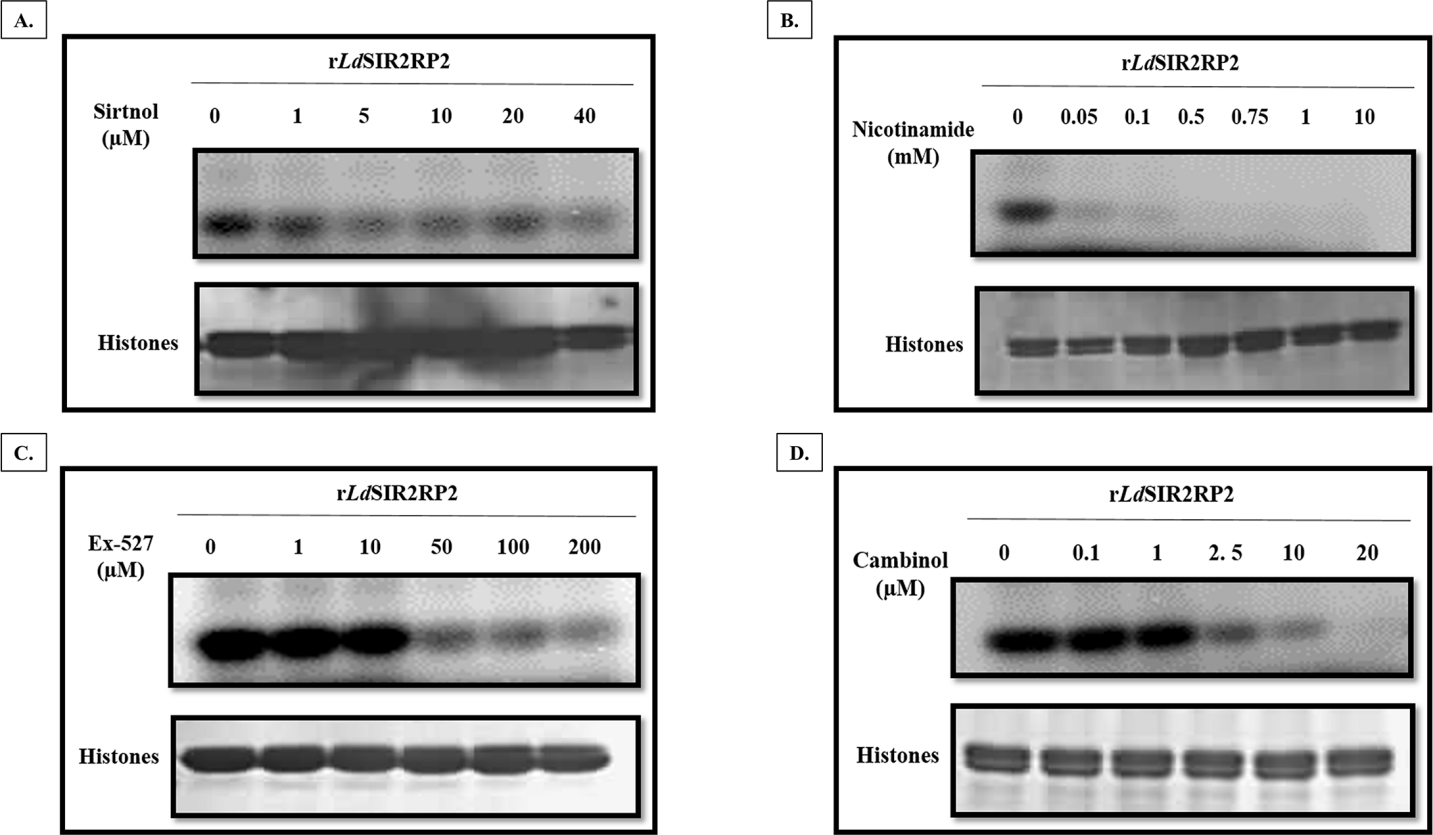
Effect of sirtuin inhibitors (A) Sirtinol, (B) Nicotinamide, (C) EX-527 and (D) cambinol, on the NAD^+^ dependent ADP-ribosyltransferase activity of recombinant *Ld*SIR2RP2. ADP-ribosyltransferase assays for r*Ld*SIR2RP2 were set up as mentioned in “materials and methods” section in the presence of indicated concentrations of each inhibitor. The top panel in each indicates the PhosphorImager visualized image and the bottom panel shows the segment of the Coomassie-stained gel corresponding to calf thymus histones shown as loading control.

Assay for the ADP-ribosyltransferase activity of *Ld*SIR2RP2 was further performed in the presence of sirtinol, NAM, EX-527 and cambinol to test their specificity and investigate the ability to inhibit the activity of recombinant *Ld*SIR2RP2. A dose-dependent inhibition of the ADP-ribosyltransferase activity of *Ld*SIR2RP2 was assessed. ADP-ribosylation assays were performed as mentioned in “materials and methods” section with varying concentrations of the inhibitors. Sirtinol did not result in inhibition of ribosylation of histones at concentrations as high as 40 μM (Fig 9A). NAM and EX-527 inhibited *Ld*SIR2RP2 activity at concentrations as low as 50 μM (Fig 9A). Out of all the four inhibitors, cambinol was the most effective in inhibiting the activity of *Ld*SIR2RP2 (Fig 9B). Concentration as low as 2.5 μM of cambinol inhibited the ADP-ribosyltransferase activity of *Ld*SIR2RP2.

## Discussion

The silent information regulator 2 (SIR2)-like family of NAD^+^ dependent protein deacetylases are highly conserved proteins from archaea to higher eukaryotes. These proteins are involved in the regulation of several functions in eukaryotic cells, including transcriptional repression, recombination, cell cycle, cellular responses to DNA-damaging agents, microtubule organisation and longevity. Sirtuins, being central to proper cellular functioning and proliferation, have been studied in protozoan parasites like *Plasmodium* and *Trypanosomes*. The parasitic sirtuins have been found to have both conserved and unique functions, which regulate a broad diversity of cellular processes, thus making them suitable drug targets for anti- parasitic therapy [16].

Three SIR2 homologs were identified in the *in silico* analysis of *Leishmania* genome; SIR2RP1, SIR2RP2, and SIR2RP3. SIR2RP1, a cytosolic sirtuin, is known to be essential for the infectivity and survival of the parasite and hence an attractive drug target for antileishmanial chemotherapy [21]. The other two mitochondrial sirtuins of *Leishmania* have not yet been characterised.

In the present study, we describe the functional role of *Ld*SIR2RP2 in *L*. *donovani*. Phylogenetic and sequence analysis reveals that *Ld*SIR2RP2 is closer to the human homolog *Hs*SIRT4, which belongs to class II of the sirtuin family. In the present study, we demonstrate that *Ld*SIR2RP2 like *Hs*SIRT4 has only NAD^+^-dependent ADP-ribosyltransferase activity. The protein was found to localise in the mitochondria of the parasite, similar to *Hs*SIRT4. Although *Ld*SIR2RP2 was not found to be essential for the survival of the parasite, the null mutants exhibited delayed growth rate and attenuated infectivity. Cell cycle analysis of the null mutant parasites revealed a G2/M block which could be a possible reason for the growth defects observed in the mutant lines. Since, *Ld*SIR2RP2 is a mitochondrial protein, analysis of the mitochondrial parameters revealed compromised mitochondria with lowered ΔΨm and hence, lesser mitochondrial ATP content of the cell. These phenotypic alterations in the Δ*LdSIR2RP2* parasites were relieved by ectopic expression of *Ld*SIR2RP2 in Δ*LdSIR2RP2*/+. Thus, deletion of the mitochondrial sirtuin *Ld*SIR2RP2 in *Leishmania* affects mitochondrial functioning leading to lowered ATP content of the cells and hence delayed growth kinetics.

*Hs*SIRT4 is a mitochondrial ADP-ribosyltransferase that inhibits mitochondrial glutamate dehydrogenase 1 activity [37]. Overexpression of SIRT4 in mammalian cells causes an increase in mitochondrial respiration, glycolysis, and glucose oxidation, but with no change in growth rate or in steady-state ATP concentrations [45]. Mitochondria, the energy provider of the cell, depends on the universal coenzyme (NAD^+^) or its phosphorylated counterpart NADP, to maintain homoeostasis within the cell [46]. In addition to participation in redox reactions, NAD^+^ acts as a versatile cellular signalling molecule through the generation of ADP-ribose (ADPR) [47]. The mitochondrial sirtuins SIRT3, SIRT4, and SIRT5 utilise mitochondrial NAD^+^ pool to regulate the activity of their targets implicated in the regulation of both glycolysis and cellular oxidative stress [46, 48] via deacetylation and mono- ADP-ribosylation.

Mono-ADP-ribosylation of proteins is a phylogenetically ancient, reversible, and covalent posttranslational modification of proteins. Both mono- and poly-ADP-ribosylation of nuclear and cytosolic proteins are known to regulate various physiological processes, such as mitosis, cellular differentiation, and proliferation, telomere dynamics, and ageing programmed necrosis and apoptosis via signalling, chromatin modification and remodelling of chromatin structure [49]. Apart from this, both, poly and mono-ADP-ribosylation modification of mitochondrial proteins is reported to have an effect on the metabolism of this organelle as well [47].

Recently, the role of mitochondrial sirtuins in parasites has been reported. It was observed that the overexpression of mitochondrial SIR2RP3 in *T*. *cruzi* led to an increase in parasite proliferation, movement, and differentiation. This was due to increased deacetylation of mitochondrial targets within the parasite [17]. Similarly, in our study, we observed a slow growth pattern and reduced infectivity upon deletion of the mitochondrial sirtuin, *Ld*SIR2RP2. The null mutants also exhibited compromised mitochondrial functioning and delayed growth kinetics. In kinetoplasts, actively respiring mitochondria are required for survival of both, the promastigotes and amastigotes [50, 51]. Thus, it can be speculated that deletion of *Ld*SIR2RP2 might have affected the activity of some of the main mitochondrial proteins which could be regulated by ADP-ribosylation, thereby affecting mitochondrial functioning in the null mutants. However, further studies are necessary to identify the specific substrates targeted by *Ld*SIR2RP2, in order to gain insight into the role of this mitochondrial sirtuin in parasite biology.

Sirtuins are known to be involved in regulation of vital cellular processes. Hence, they have been proposed as promising targets for the development of anti-parasitic drugs [42, 52, 53]. Here, the efficacy of known sirtuin inhibitors; sirtinol, nicotinamide, Ex-527, and cambinol, on the growth of the WT and genetically manipulated parasites was determined. Except for sirtinol, all the other three inhibitors were more effective in inhibiting the growth of Δ*Ld*SIR2RP2 parasites than the WT. This increased susceptibility of the inhibitors was relieved by ectopic expression of *Ld*SIR2RP2. The concentrations tested had no significant effect on the host cells, indicating a selectivity of these inhibitors for the parasitic sirtuins than for the host sirtuins. While these inhibitors show specificity towards the ADP-ribosyltransferase activity of *Ld*SIR2RP2, we cannot rule out the off-target effect of these inhibitors on the other two sirtuins. Earlier studies have demonstrated that NAM inhibits recombinant *Li*SIR2RP1 which is both a deacetylase and ADP-ribosyltransferase (15). Furthermore, overexpression of either of these sirtuins, *Tc*SIR2RP1 and *Tc*SIR2RP3 protected the parasite from the effect of cambinol and NAM (17).

With increasing drug resistance, toxicity and the cost of the available chemotherapeutic agents for the treatment of Leishmaniasis, the development of new leishmanicidal drugs and the search for new targets is required. The peculiar differences between the parasite and mammalian mitochondria, as well as unique characteristics of parasite mitochondria, makes mitochondrial proteins as good drug targets. Several new studies involving inhibitors like; Benzophenone-derived bisphosphonium salts [54], artemisinin [55], chalcones, including licochalcone A [56], Tafenoquine [57], luteolin and quercetin [58]; indicate the essential role of mitochondrial biology in the survival of the parasite.

Here, we have attempted to characterise a mitochondrial sirtuin, which is involved in maintaining the mitochondrial homoeostasis. *Ld*SIR2RP2 deletion resulted in reduced growth and virulence of the parasite. Known sirtuin inhibitors were able to inhibit the growth of the parasite. The inhibitors also showed inhibitory effect on the enzymatic activity of recombinant *Ld*SIR2RP2. However, the pleiotropic effect of these inhibitors on the other two parasitic sirtuins, *Ld*SIR2RP1, and *Ld*SIR2RP3, cannot be ruled out. Thus, developing a specific inhibitor to target *Ld*SIR2RP2 alone or in combination with the available chemotherapeutic agents could provide a better rationale for the treatment of Leishmaniasis.
